# Persistence of Diarrheal Pathogens Is Associated with Continued Recruitment of Plasmablasts in the Circulation

**DOI:** 10.1155/2012/279206

**Published:** 2012-01-19

**Authors:** Anu Kantele

**Affiliations:** ^1^Division of Infectious Diseases, Department of Medicine, Helsinki University Central Hospital, Aurora Hospital, Building 5, 3rd floor, POB 348, 00029 Helsinki, Finland; ^2^Department of Bacteriology and Immunology, Haartman Institute, University of Helsinki, 00014 Helsinki, Finland; ^3^Institute of Clinical Medicine, University of Helsinki, 00014 Helsinki, Finland

## Abstract

Intestinal antigen encounter leads to recirculation of antigen-specific plasmablasts via lymphatics and blood back to the intestine. Investigating these gut-originating cells in blood provides a less invasive tool for studying intestinal immune responses, with the limitation that the cells disappear from the circulation in two weeks. No data exist on situations where pathogens persist in the intestine. Patients with *Salmonella, Yersinia,* or *Campylobacter* gastroenteritis and volunteers receiving an oral typhoid vaccine were assayed for plasmablasts specific to each subject's own pathogen/antigen weekly until the response faded. In vaccinees, plasmablasts disappeared in two weeks. In gastroenteritis, the response faded 2-3 and 3–7 weeks after the last positive *Salmonella* or *Yersinia* stool culture. Even in symptomless patients, pathogens persisting in the intestine keep seeding plasmablasts into the circulation. Assaying these cells might offer a powerful tool for research into diseases in which persisting microbes have a potential pathogenetic significance.

## 1. Introduction

The intestine represents the largest immunological tissue in the body and carries the majority of all lymphocytes [1, 2]. Pathogens encountered in the intestine activate antigen-specific lymphocytes in Peyer's patches, and these cells migrate to mesenteric lymph nodes and further via lymphatics and blood to the intestinal lamina propria as effector lymphocytes [2–6].Consistent with this recirculation of activated intestinal lymphocytes, antigen-specific effector lymphocytes have been found in the circulation after intestinal antigen encounter both after oral [7–11] and rectal  [10, 12] vaccinations and in intestinal infections [13–15].

The mechanisms underlying this recirculation of activated intestinal lymphocytes have been a subject of extensive research. It has been shown that dendritic cells in the intestine present the antigens to lymphocytes in Peyer's patches and program these cells to express a set of receptors determining their later migratory behavior [2–6]. Next, these activated lymphocytes migrate to mesenteric lymph nodes and return to the mucosal sites with the help of lymphatics and blood. This return once appeared to occur randomly with circulating blood, but, in fact, it exhibits marked tissue selectivity at the final stage of homing from blood through the endothelium into the tissues. This homing is a multistep process requiring an interaction of lymphocyte surface molecules recognizing their ligands distributed in a tissue-specific manner in the body [2–6]. Lymphocytes homing to the intestine express both CCR9 [1–3, 16], a chemokine receptor mediating homing to the small intestine, and *α*_4_ *β*_7_ [1–3, 17], a gut-specific homing receptor (HR) that recognizes Mad-CAM1 on the endothelial venules of the intestine. The majority of gut-originating antigen-specific plasmablasts found in the peripheral blood after oral vaccination [9–11] or in intestinal infections [14] have been shown to express the gut HR, *α*_4_ *β*_7_, implying a preferential homing of these cells to the intestinal lamina propria.

Research into intestinal immune responses in the human gut has been hampered by the instability of antibodies in secretions and, to an even greater extent, by ethical restrictions. Investigation of gut-originating plasmablasts in the peripheral blood circumvents some of these problems. Plasmablasts (preplasma cells) are close to end-stage cells of the B-cell lineage representing only a minor part of all circulating B cells: recently activated and developed into effector cells, they constitute the part that actively secretes antibodies while being on their way to settling down in the target tissues as plasma cells. Plasmablasts recently activated in the intestine are identified in the circulation as antibody-secreting cells (ASCs) with a spontaneous secretion of antibodies against intestinally encountered antigens [7–15]. Studies with oral vaccines have shown that these cells can be caught from the peripheral blood 3–5 days after the original antigen encounter, they peak in number around day 7 and disappear within two weeks [7, 8, 18], consistent with their homing back to the mucosa. Catching gut-originating effector lymphocytes from the circulation has proved a useful tool in studying mucosal immune responses to both oral vaccines [7–11] and intestinal infections [13–15]. However, a major restriction to this approach is the transient nature of the response: the actual window for catching the cells is no longer than a few days. The kinetics has been explored with oral vaccinations [7, 8, 18] where the exact time of antigen encounter is known and the vaccine antigen persists in the intestine for only a short time. The present study investigates the response in natural infections where the pathogen persists in the intestinal milieu for a longer period of time. This is of interest also in view of diseases in which pathogen persistence plays a role in the pathogenesis of the disease.

## 2. Methods

Patients with diarrhea and volunteers receiving an oral typhoid vaccine were studied for circulating plasmablasts specific to the pathogen isolated from their own stool sample or to the vaccine antigen, respectively. The kinetics of the response was studied along with determinations of isotypes. In order to confirm the intestinal homing commitment of these migrating cells, the homing profile was determined in a subgroup of volunteers in both groups.

### 2.1. Patients

23 patients attending the Central Hospital of Central Finland or the Helsinki University Central Hospital and six healthy volunteers were enrolled in the study. Informed consent was obtained from each patient/volunteer before participation. The study was conducted according to the principles stated in the Helsinki Declaration on human experimentation, and the study protocol was reviewed and approved by the Ethics Committees of the participating hospitals.

22 patients (12 women, 10 men, aged 36–63 years) had bacterial diarrhea, diagnosed on the basis of watery stools and a pathogen isolated from stool samples. One patient had no diarrhea but was examined because of her spouse's positive *Salmonella *culture: her stool culture proved positive for the same pathogen. In her case, the day of the first positive stool sample was defined as day 0, while, for all others, day 0 indicates the day of the first symptoms. None of the patients had a known previous history of diarrhea caused by a bacterial pathogen, nor had they been diagnosed with immune deficiencies or other significant underlying diseases. Six healthy volunteers (all women, aged 24–47), who had not received the typhoid vaccination previously, were each given the oral *Salmonella typhi *Ty21a vaccine (Vivotif, Crucell, Switzerland) in three doses two days apart, according to the manufacturer's instructions. For vaccinees, the day of the first vaccine dose was marked as day 0.

### 2.2. Collection of Blood Samples, Isolation of PBMC, and Preparation of Antigen

For patients with diarrhea, first blood samples for ASC analyses were drawn 6–33  days after the onset of the symptoms, and, for vaccinees, a series of blood samples was drawn 0, 5, 7, 9, 12, 14, and 16 days after the oral typhoid vaccination and then, in both groups, weekly for as long as the ASCs were detected or the pathogen could be recovered from the stool samples, and the patient was available. Mononuclear cells were isolated from heparinized blood by Ficoll-Paque density-gradient centrifugation as described previously [8] and adjusted to a concentration of 2 × 10^6^ cells/mL. HR expressions were determined in the first blood sample drawn in five patients with diarrhea and on day seven after vaccination in five vaccinees.

Bacterial strains identified in the stool samples were grown on nutrient agar plates and formalin-killed as described previously [13, 14, 19, 20]. The concentration of the bacterial suspension was adjusted to 10^8^ bacteria/mL.

### 2.3. Separation of Receptor-Negative and Receptor-Positive Cell Populations

The separation of the cells into receptor-negative and receptor-positive populations has been described earlier [9, 14, 21]. Briefly, the cells were incubated with the monoclonal antibodies anti-*α*_4_ *β*_7_ (ACT-1) (Millennium Pharmaceuticals, Cambridge, MA), anti-L-selectin (Leu8) (Becton-Dickinson), or anti-CLA (HECA-452) (received from Dr. Sirpa Jalkanen, University of Turku, Finland), washed twice, and incubated with Dynal M-450 magnetic beads coated with sheep anti-mouse IgG (Dynal, Oslo). The beads with the attached cells were separated from the suspension by applying a magnet outside the test tubes and the supernatants with the receptor-negative cells collected. The receptor-positive cells attached to the beads were collected. Both the receptor-positive and receptor-negative cell populations were immediately analyzed with the enzyme-linked immunospot (ELISPOT) assay for numbers of ASC.

The efficiency of the cell separations was checked in pilot experiments as described previously [9, 14].

### 2.4. Assay of Specific Antibody-Secreting Cells

The total population of PBMC and the receptor-positive and receptor-negative cell populations were each assayed for specific ASC with ELISPOT: the assay of specific ASC has been described previously [8, 13, 14, 19, 20]. In brief, for patients with diarrhea, 96-well microtiter plates (Maxisorp, Nunc. Denmark) were coated with a whole-cell preparation of formalin-killed pathogen isolated from the stool sample of the same patient (see above) or, for vaccinees, with a preparation formalin-killed *Salmonella *strain SL2404 carrying the O-antigen 9 and 12 similarly to the vaccine strain Ty21a. The cells were incubated in the wells, and antibodies secreted during this time detected with alkaline phosphatase-conjugated anti-human IgA (Sigma-Aldrich, Mo, USA), IgG (Sigma-Aldrich), and IgM (SouthernBiotech, Birmingham, AL, USA) followed by application of substrate (bromo-4-chloro-3-indolyl phosphate p-toluidine salt; Sigma-Aldrich) in melted agarose. Specific ASCs were enumerated by counting the spots in the wells in a light microscope. A response was defined as >2 specific ASC/10^6^ PBMC in at least one sample.

### 2.5. Statistics

The numbers of ASC were calculated as geometric means ± SEM. The proportions of the receptor-positive ASC were calculated as follows: % of receptor-positive cells among ASC = (100 X the number of ASC in receptor-positive population) ÷ (the sum of the number of ASC in receptor-positive and receptor-negative populations). Statistical comparisons were carried out using Student's *t*-test. Results of statistical analyses were considered significant when *P* < 0.05.

## 3. Results

### 3.1. Pathogens and Symptoms

In patients with gastroenteritis, *Salmonella enteritidis *was grown as a pathogen in stool culture in 10/23 cases, *Yersinia enterocolitica *in 7/23, *Campylobacter jejuni *in 2/23, and *S. stockholm, S. bradford, S. emek, *and *S. typhimurium *each in one case. In 12/23 patients, only one positive stool sample was obtained, and the second one taken 1-2 weeks later was negative. In 11/23 patients, 2–11 positive stool samples were collected. 12/23 patients had symptoms during the first sampling, and 10/23 of them until 1–5 weeks after it. In 3/23, patients the symptoms had faded away one week, and in 8/23 patients in 2–4 weeks before the first blood sample.

### 3.2. General Characteristics of the Antigen-Specific Plasmablast Response

**Figure 1:**
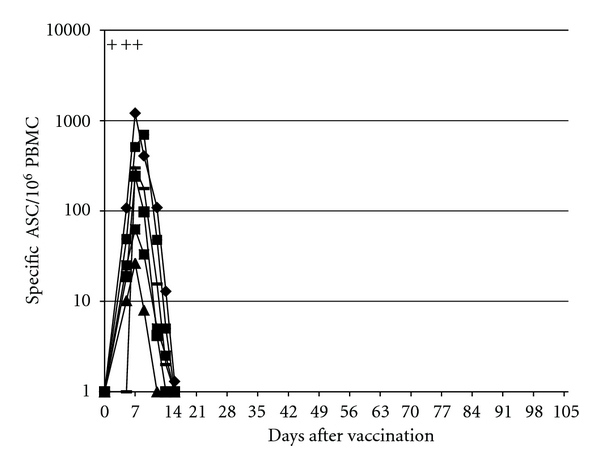
Numbers of vaccine antigen (O9,12)-specific circulating plasmablasts identified as antibody-secreting cells/10^6^ PBMC (IgA- + IgG- + IgM) in six volunteers vaccinated with the live oral *Salmonella typhi *Ty21a vaccine. Each volunteer received one vaccine dose on days 0, 2, and 4 (the vaccination days are indicated with “+”). To determine the kinetics of the response, the numbers of plasmablasts were determined on several days for as long as ASCs were found in the samples. The values of each individual are connected with a line.

All six volunteers vaccinated with Ty21a showed a vaccine-antigen-specific ASC response similar to that in our previous studies [8, 12] (Figure 1).

**Figure 2:**
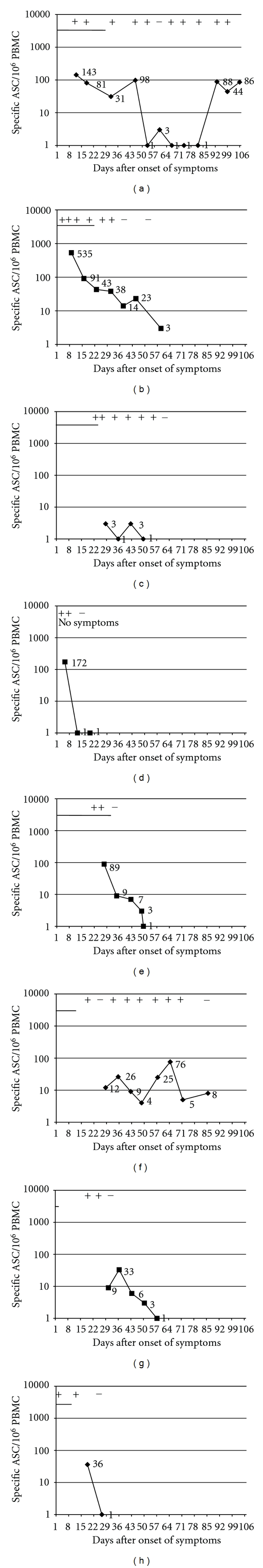
Relation of pathogen-specific circulating plasmablasts (black curve) with symptoms (black horizontal line) and findings in stool samples (+ or −) in eight patients with gastroenteritis caused by *Salmonella enteritidis*. To determine the kinetics of the response, the numbers of plasmablasts were assessed on several occasions as long as they were found in the samples or the pathogen could be isolated from the stool samples, and the patient was available. The plots represent each data from one individual. The day of the onset of the symptoms was marked as day 0.

**Figure 3:**
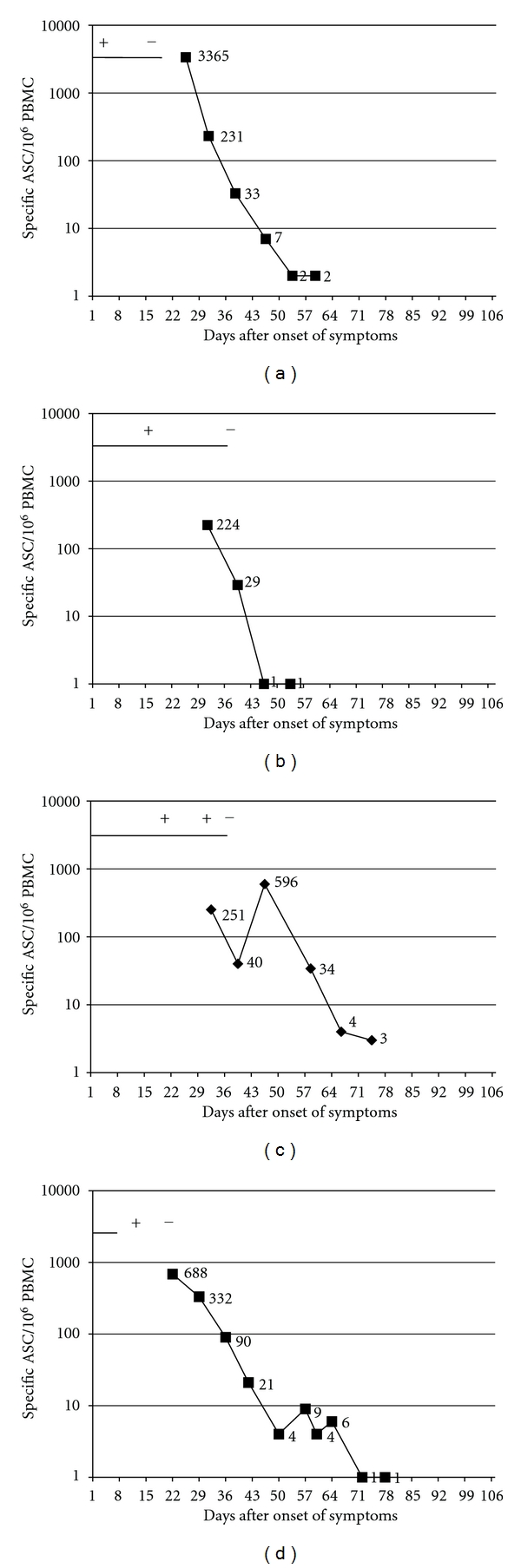
Relation of pathogen-specific circulating plasmablasts (black curve) with symptoms (black horizontal line) and findings in stool samples (+ or −) in four patients with gastroenteritis caused by *Yersinia enterocolitica*. The plots represent each data from one individual. The day of the onset of the symptoms was marked as day 0.

A response of circulating pathogen-specific plasmablasts was found in 8/10 patients with *Salmonella enteritidis* (Figure 2). In one patient, there was no response despite a positive stool sample one week before (symptoms started 22 days earlier). In one patient, the blood sample was obtained only four weeks after the last positive stool sample and last symptoms. Circulating ASCs were detected in 5/7 patients with *Yersinia enterocolitica* (Figure 3). In one patient with a clear response, no further follow-up samples could be obtained. No ASCs were found in 2/7 patients: in both of these cases, the sample was drawn one week after the symptoms had faded and four weeks after the last positive stool sample.

**Figure 4:**
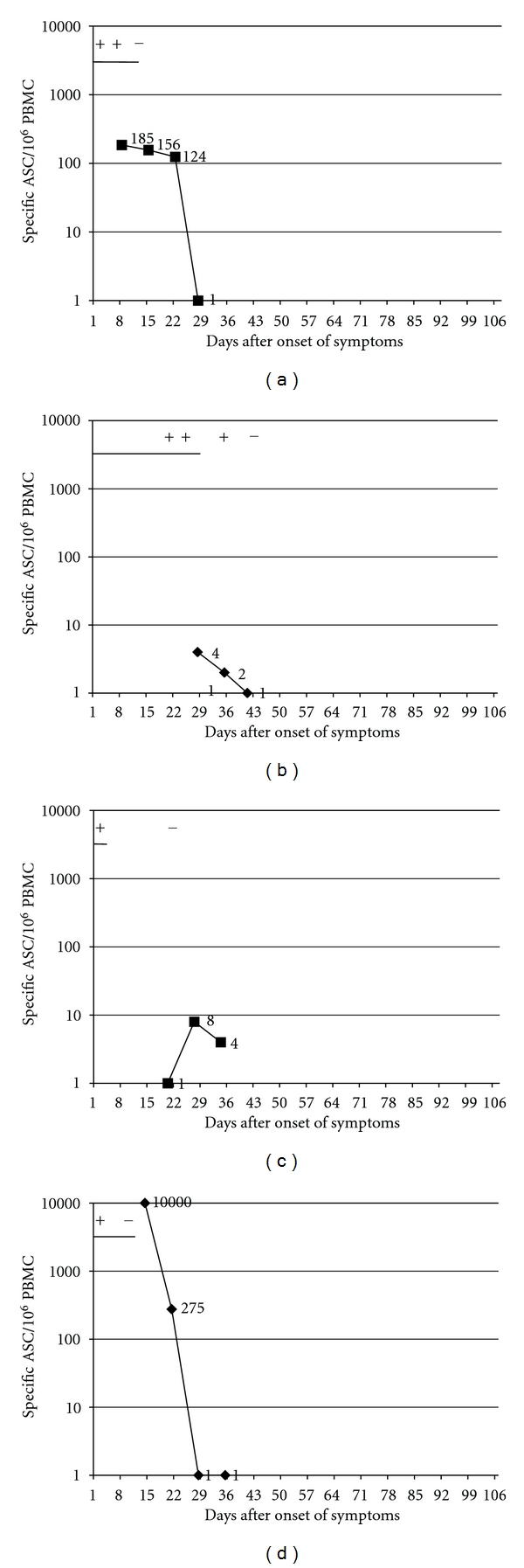
Relation of pathogen-specific circulating plasmablasts (black curve) with symptoms (black horizontal line) and findings in stool samples (+ or −) in four patients with gastroenteritis caused by (a) *Salmonella typhimurium*, (b) *S. emek*, (c) *S. bradburg,* and (d) *Campylobacter jejuni*. The plots represent each data from one individual. The day of the onset of the symptoms was marked as day 0.

Patients with *S. bradford, S. emek, *and *S. typhimurium *as pathogens had a response (Figures 4(a)–4(c)). The patient with *Salmonella stockholm *did not show a response even though the samples were drawn one week after positive stool culture and last symptoms. One patient with *Campylobacter jejuni *had a vigorous response (10 000 ASC/10^6^ PBMC) (Figure 4(d)), while the other one showed no response; her blood sample was drawn three weeks after the last positive stool sample.

**Figure 5:**
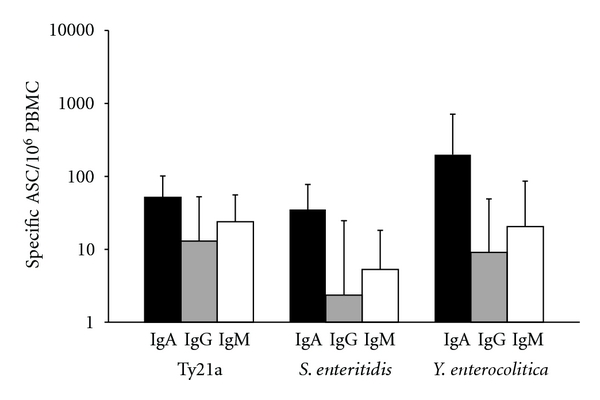
The isotype distribution in the peak of the specific plasmablast response in volunteers vaccinated with the oral live *Salmonella typhi* Ty21a vaccine (*n* = 6) and in patients with gastroenteritis caused by *Salmonella enteritidis *(*n* = 8) or *Yersinia enterocolitica *(*n* = 5). The data are given as geometric means of ASC /10^6^ PBMC ± SEM.

Among the vaccinees, IgA-ASC predominated in three volunteers, IgG in two, and IgM in one. The geometric mean of the peak of the responses (IgA + IgG + IgM-ASC) was 215 ± 184/10^6^ PBMC. The isotype distribution is shown in Figure 5. 

Out of all patients, the response was dominated by IgA in 15/17 cases and IgM in 2/17 cases (one with *S. typhimurium *and one with *Yersinia enterocolitica*). The geometric mean of the peak of the responses (IgA + IgG + IgM-ASC) was  64 ± 70/10^6^ PBMC in patients with *S. enteritidis *and 459 ± 614/10^6^ PBMC with *Yersinia enterocolitica*. The isotype distributions are shown in Figure 5.

### 3.3. Kinetics of the Antigen-Specific Plasmablast Response

In the vaccinees, the response peaked on day 7 and declined thereafter. No samples were drawn after day 16, since the response had faded away/was negligible already on that day in all volunteers (Figure 1).

In patients with gastroenteritis, the numbers of antigen-specific ASC were followed weekly for as long as the response persisted or the stool culture remained positive, and the patient was available. The highest number of ASC was in 12/16 cases found in the first sample drawn, that is, soon after the beginning of symptoms and after that the magnitude of the response appeared to fluctuate until it faded away (Figures 2–4). The numbers of weekly blood samples drawn varied from one till up to 12 (Figures 2–4). The fading of ASC appeared to be associated with the stool samples turning negative, rather than the subsiding of the symptoms, as the ASC response was prolonged in some volunteers with persisting positive stool cultures even after the symptoms had subsided (Figures 2(a), 2(c), 2(f)). The response appeared to fade away faster after *Salmonella *(2-3 weeks) (Figure 2) than after *Yersinia *infection (3–7 weeks) (Figure 3). 

### 3.4. The Expression of HR on Antigen-Specific Plasmablasts

The expressions of various HR on ASC specific to the diarrheal pathogen or the vaccine antigen were studied in five patients with *Salmonella *diarrhea and in five vaccinees, respectively. In the diarrhea group, the proportion *α*_4_ *β*_7_ + ASC among all ASC was 94 ± 6%, L-selectin + ASC 46 ± 24%, and CLA + ASC 4 ± 4%. Among vaccinees, the respective figures were 97 ± 2%, 24 ± 18%, and 1 ± 2%. These data did not reveal any statistical difference in the HR expressions between diarrhea patients and vaccinees but are consistent with the intestinal homing profile of the migrating cells in both groups.

## 4. Discussion

Investigation of gut-originating plasmablasts from samples of peripheral blood during their recirculation serves as a valuable, less invasive tool for studying intestinal immune response in humans. Instead of examining memory B cells, this approach centers on gut-directed plasmablasts cells spontaneously secreting antibodies against antigens they have encountered recently, that is, the method measures an ongoing intestinal immune response. The transient appearance of these cells in the circulation, which allows only a few days for actual sampling, has posed a severe limitation to this approach. The present study is the first to focus on the kinetics of this response during a natural infection in which the pathogen can persist in the intestine for a longer period of time. It shows that the previously presumed kinetics does not apply to natural infections: if the pathogens persist in the intestine, the actual time frame for sampling can be significantly extended.

### 4.1. Comparison to Serum Antibody Responses

Assessment of plasmablasts has significant advantages as compared to serum antibody assays. Firstly, plasmablast response measures an immune response to antigens encountered recently, whereas serum antibodies may remain elevated throughout life even if the antigen was encountered decades ago [22]. Secondly, plasmablast assay allows an assessment of the response to each individual's own pathogen. Thirdly, plasmablast response has proved significantly more sensitive than serum antibodies when measuring humoral immune response to intestinal antigen encounter [8]. Our previous studies have shown that shortly after oral vaccination, when antibodies are presumed to exist in intestinal secretions, serum antibodies even fail to rise at all upon intestinal antigen encounter, despite a simultaneous significant plasmablast response [23]. This describes the independent nature of systemic and mucosal immune systems and further stresses the role of plasmablast instead of serum antibody measurements when assessing intestinal immune responses: plasmablasts represent intestinal immune response, whereas serum antibodies are mainly produced in the bone marrow. Serum antibodies remaining fairly constant for long periods of time do not provide any information as to whether the pathogen persists in the intestine or the pathogen has been cleared. Up until now, it has been assumed that exploring plasmablasts would not provide such information either, as the cells were thought to be found in the circulation for two weeks only. The continued recruitment of plasmablasts in the present study suggests that the use of the assay of specific plasmablasts can be extended and even applied to the evaluation of antigen persistence in the intestine.

### 4.2. Significance of the Continuous Recruitment of Plasmablasts

Continuous recruitment of plasmablasts appears to be a means of enhancing the immune response to a pathogen the body has not succeeded in expelling. The generally lower numbers of plasmablasts recruited as the time passes and the even negligible numbers seen occasionally in the later course of the response may be a reflection of antibody responses in the intestine already working against the pathogens. However, such fluctuations in the magnitude of the response with occasional negligible numbers of plasmablasts strongly suggest that a single negative sample does not indicate that the pathogen no longer persists, and therefore, multiple sampling may be required. The highest numbers of plasmablasts are found early in the course of the response. Previous to this we have, in fact, shown that if an oral vaccine is given in six instead of three doses, the plasmablast response is prolonged till day 22, and even if no clear peak can be seen, the response is at its highest on day 7 [24]. The early peak in the response means that (possible) comparisons in response magnitude between two groups should be carried out on the basis of samples drawn at early stages of the disease, while, in other samples, a qualitative comparison is mainly feasible. It would be interesting to see if chronic carriers of intestinal pathogens (e.g., Salmonella) still have an ongoing recruitment of plasmablasts, as the pathogen can in such cases be regarded to have become part of their regular intestinal microbiota. As to the disappearance of ASC, it is not possible to draw any definite conclusions. In the present data, however, the response seemed to fade away faster after *Salmonella* (2-3 weeks; Figures 2 and 4) than after *Yersinia* infection (3–7 weeks; Figure 3). As opposed to infections with real multiplying pathogens, the Ty21a vaccine strain is supposed to survive in the intestine only for one or two days. Accordingly, data comparing responses after three versus six doses of Ty21a [24] suggest that in cases with nonreplicating antigens, the plasmablasts disappear from the circulation approximately 8–10 days after the last day of antigen encounter.

Notably, even if the present study focuses on plasmablast response after *intestinal *antigen encounter, a recirculation of antigen-specific plasmablasts also occurs after *nonintestinal* encounter [9, 25–27], for example, in infections at other mucosal sites [10, 19, 20, 28] and after parenteral vaccinations [9, 14, 25–27, 29, 30]. As evidenced by their HR profiles, these plasmablasts are trafficking to nonintestinal sites. Thus, it appears that assaying these cells could be applied to assess persistence of pathogens at nonintestinal sites, too.

## 5. Conclusion

In conclusion, the recruitment of pathogen-specific plasmablasts in the circulation in gastroenteritis has proved less transient than previously reported. Instead, the recruitment seems to continue as the pathogen persists in the intestine, albeit at a lower or, occasionally, even negligible level, indicating that repeated sampling may be necessary. Continued recruitment of plasmablasts in the circulation not only reflects a continuous stimulation of the immune system but also carries potential for assessing immune response to persisting antigens that are suspected to be of significance in the pathogenesis of a disease.

## Abbreviations

ASC: Antibody-secreting cell
CCR: Chemokine receptor
HR: Homing receptor.
